# Long-term patient-reported outcomes after plication of rectus diastasis and simultaneous herniorrhaphy with HELP abdominoplasty

**DOI:** 10.1007/s10029-025-03408-6

**Published:** 2025-07-05

**Authors:** Julia Saxen, Hilkka Peltoniemi, Tiina Jahkola, Jaana Vironen, Reetta Tuominen

**Affiliations:** 1 Department of Plastic Surgery, Helsinki University Hospital and University of Helsinki, Helsinki, Finland; 2 Abdominal Center, Helsinki University Hospital and University of Helsinki, Helsinki, Finland; 3 Eira Hospital, Helsinki, Finland

**Keywords:** Abdominal rectus diastasis, Abdominoplasty, Umbilical Hernia, Epigastric Hernia

## Abstract

**Purpose:**

Midline hernias are common, and when associated with abdominal rectus diastasis, hernia guidelines recommend correction using mesh techniques. We present a retrospective series of patients with primary midline hernias and post-pregnancy moderate or severe abdominal rectus diastasis, who were operated using a comprehensive surgical approach without mesh.

**Methods:**

We previously described the HELP (Hydrodissection-Assisted Extended Lateral Plication) abdominoplasty technique for rectus diastasis repair, with or without a midline hernia. In this study, patient records from 2013 to 2018 were reviewed, and patients with a midline hernia who underwent the HELP abdominoplasty were recruited for a retrospective analysis.

**Results:**

Seventeen patients were successfully contacted. The mean diameter of the umbilical hernia defect was 13.6 mm (5–30 mm), and 7.7mm (5–20 mm) in epigastric hernias. The mean follow-up period was 5.2 years. None of the patients reported a recurrence of diastasis or of hernias. The overall complication rate was 11.8%.

**Conclusion:**

HELP abdominoplasty appears to be a reliable surgical treatment with a low complication rate for normal-weight women with post-pregnancy moderate to severe rectus diastasis and concomitant small primary hernias. In these cases, the entire damaged fascia should be repaired, and mesh correction is not always necessary.

## Introduction

The linea alba is the fusion of aponeuroses between the two rectus abdominis muscles, and a pathological increase in its width is diagnosed as abdominal rectus diastasis (RD) [1, 2]​. RD can result from any condition that increases intra-abdominal volume, commonly due to aging, obesity and pregnancies [3]​​. The anterior abdominal wall biomechanically influences lumbar spine movement and stability, as well as abdominal strength [4, 5]​​. RD has been shown to contribute to back pain and core instability in post-partum women; in such cases, surgical correction of the defect can improve quality of life [6, 7]​​.

RD and midline hernias are closely related: RD predisposes patients to midline hernias and is a significant risk factor for hernia recurrence. Mesh techniques are commonly recommended for correction of these hernias [8–11]​​. Due to the variety of different patient groups’ physiological tissue qualities, the technique used for the herniorrhaphy of women with post-pregnancy RD should most likely be non-MESH-augmented. The senior surgeon of this study, H.P., has utilized the HELP (Hydrodissection-Assisted Extended Lateral Plication) abdominoplasty technique for over two decades in normal-weight female patients with symptomatic post-pregnancy RD and a protruding abdominal wall, with or without a concomitant midline hernia. In this study, we describe the long-term outcomes of the HELP abdominoplasty in patients with RD and small and medium size midline hernias, with up to 8 years of follow-up.

## Methods

### Patients and data collection

HELP (Hydrodissection-Assisted Extended Lateral Plication) abdominoplasty technique is described in a prospective patient series [12]​​. This study recruited patients operated on by co-author Dr. Peltoniemi at Tilkka Hospital between March 2013 and October 2018. The study population comprises of patients who financed their treatment either through out-of-pocket payments or via insurance coverage. Twenty patients met the inclusion criteria, having undergone HELP abdominoplasty for a midline hernia and concomitant diastasis recti correction. None of the patients included in the study had a history of massive weight loss. Seventeen of the twenty patients were successfully contacted via telephone or email, and their perceptions of the herniorrhaphy and RD repair were assessed using a Likert scale [13]​. Three patients could not be reached due to outdated contact information. All contacted patients were willing to participate. The questionnaire is detailed in Table 1, and descriptive results are reported in Table 2. The study was approved by the Regional Ethics Review Board at Helsinki University Hospital (1815/2021). Data collection occurred, on average, 5.2 years postoperatively (2.2–7.8 years). Demographic data included body mass index, the number of pregnancies, IRD, and hernia size.

**Table 1:** Retrospective questionnaire

	Mean	95% CI
Patients	17	
Before surgery, you had a midline hernia. If you assess you abdominal wall now, do you consider that the operative result has preserved and you do NOT have a hernia recurrence at the moment? (p)	4.9	[4.82, 5.06]
Before surgery, you had abdominal rectus diastasis. If you assess you abdominal wall now, do you consider that the operative result has preserved and you do NOT have a diastasis recurrence at the moment? (p)	4.9	[4.82, 5.06]
If you had back pain before the operation, did the operation improve the situation? (p)	4.9	[4.65, 5.07]
If you had core stability problems (standing long periods, perform sports you used to perform, difficulties in sit ups etc.) before the operation, did the operation improve the situation? (p)	4.9	[4.67, 5.06]
Are you satisfied with the operation overall? (p)	5	[5]
How much has you weight changed after the operation? (kg)	0–5	
Scoring algorithm		
Likert scale		
1p	Strongly disagree	
2p	Disagree	
3p	Neither agree nor disagree	
4p	Agree	
5p	Strongly agree

**Table 2:** Demographics and characteristics

Number of patients	17	
Body mass index, mean kg/m2 (range)	22.8	(20.1–27.9)
Pregnancies mean (range)	2	(1–4)
Inter-rectus distance, mean cm (range)	5.2	(2.5–8)
Plication, mean cm (range)	7.7	(4.5–13)
Epigastric hernia size, mean mm (range)	6	(5–20)
Umbilical hernia size, mean mm (range)	13.6	(5–30)
Complications, no. (%)	2/17	(11.8%)
Follow-up, years mean (range)	5.5	(2.2–7.8)

### Surgical technique

#### HELP abdominoplasty

HELP abdominoplasty was performed as previously described in more detail [12]​. The operation is conducted under epidural anesthesia and includes hydrodissection of tissues. The technique consists of two layers of sutures. The first layer plicates the diastasis using non-absorbable, braided, interrupted sutures in a figure-of-eight pattern. The second layer completes the plication using an absorbable barbed running suture, which also functions to conceal the suture material and minimize its palpability through the skin. The plication extends beyond the medial borders of the rectus muscles in cases of lateral laxity​ [14]​. An important aspect of the HELP plication is addressing overall laxity by extending the plication laterally over the rectus sheath as far as necessary. Abdominoplasty and the removal of any excess skin are performed as usual. The detected hernias are dissected, reduced and the orifice is sutured with slowly resorbable sutures.

## Results

Retrospective patient demographic data is presented in Table 2. The mean BMI was 22.8 kg/m^2^ (20.1–27.9 kg/m^2^), with three participants exceeding a BMI of 25.0 kg/m^2^. Two patients (3R and 17R) had previously undergone midline hernia repair with a ventral patch and experienced hernia recurrence. Re-herniorrhaphy was performed using the HELP method. Inter-rectus distance (IRD) values were measured intraoperatively with calipers. The mean IRD in this series was 5.5 cm (range 2.5–8 cm). Twelve of the seventeen patients had a wide diastasis (D3, > 5 cm), four had a moderate diastasis (D2, 3.0–5.0 cm), and one had a mild diastasis (D1, < 3 cm)​ [2]​. The mean umbilical hernia size was 13.6 mm (5–30 mm), and the mean epigastric hernia defect size was 6 mm (5–20 mm). Four of seven patients (6R, 8R, 13R, and 17R) with an epigastric hernia had multiple small hernias along the stretched midline, carrying a risk of developing larger hernias due to the fragile and damaged linea alba.

Complications recorded in this retrospective series included one bedside hematoma, which was evacuated under local anesthesia a few hours after the initial operation in patient 4R (Clavien-Dindo Class IIIa). Also, a mild infection of the navel area was reported and treated with local antibiotic ointment in patient 8R (Clavien-Dindo Class I). Thus, the total complication rate was 11.8%.

In the retrospective analysis, all participants were interviewed regarding their perceptions of hernia recurrence, RD recurrence, the effect of surgery on back pain and core stability, and overall satisfaction with the HELP abdominoplasty (Table 1). All parameters were assessed using a Likert scale. All patients were highly satisfied with the operation overall. Functional outcomes were good: 16 of 17 patients strongly agreed that there was no hernia recurrence (five points), the remaining one patient also agreed there was no recurrence (four points). The same results were observed for the RD recurrence. Fourteen of the seventeen patients reported back pain before the operation, and postoperative assessments were favorable: two patients scored four points, and twelve scored the maximum of five points. Fifteen of the seventeen patients reported preoperative core stability issues. Postoperatively, two patients scored four points, and thirteen scored five points, indicating a clear benefit from the surgery.

## Discussion

Abdominal rectus diastasis (RD) is an emerging topic in the field of abdominal wall defects and treatment [15]​​. The management of a plain RD has been controversial, with treatment indications varying in public healthcare. Due to the poor and thin quality of the linea alba in RD patients, small hernias are common [10]​​. While there is a lack of clear guidelines regarding the use of mesh for reinforcing rectus diastasis (RD) repair [16], the use of mesh is typically recommended for umbilical and midline hernia repair in conjunction with RD [11, 17, 18]. Especially when the inter-rectus distance (IRD) exceeds 5 cm [9, 19, 20], the need for mesh should be evaluated​. There is some evidence that ventral hernias up to 3 cm with concomitant RD can be effectively repaired using suturing techniques contrary to the EHS guidelines, which recommend mesh repair for hernias over 1 cm in diameter [21, 22]. There is an abundance of Endoscopic Onlay Repair (ENDOR) methods for subcutaneous endoscopic onlay repair of ventral hernias with anterior plication of the RD that do not address the excess skin generated by the underlying fascia plication [2, 23]​. In many patients his abundance of skin must be assessed and possibly excised.

The fascial tissue condition differs between obese patients and normal-weight or slim post-pregnancy RD patients. Unlike obese patients, normal-weight or slim post-pregnancy RD patients have an abundance of fascial tissue but lack excess intraperitoneal content to stretch the abdominal wall. Therefore, the risk of recurrent midline hernia might be lower in the RD patient group after comprehensive midline correction.

The HELP abdominoplasty is designed for normal-weight women with post-pregnancy abdominal wall issues. The outcomes were favorable, with no hernia or diastasis recurrences and high patient satisfaction. In this retrospective series of seventeen RD patients, two had previously undergone mesh-augmented herniorrhaphy but developed hernia recurrence. This highlights the importance of addressing the entire damaged midline, not just the hernia, in normal-weight patients with combined hernia and RD [11]​. Re-operation using the HELP abdominoplasty after ventral mesh placement can be challenging, as the fascia is locked in a wide position, resisting plication.

Previous studies have shown that the short-term complication rate after open umbilical hernia repair ranges from 2.5% to 5.4%, and 0% to 5.4% after laparoscopic repair (wound infection 5.4%/1.9%, wound dehiscence 2.5%/0%, hematoma 4%/0.9%, seroma 4.3%/5.4%) [24]​​. Many studies report Clavien-Dindo grade 3 and higher complications leading to readmissions [25, 26]​. The complication rate of abdominoplasty as a sole surgical procedure is approximately 0–25% (wound infection 0–20.4%, wound dehiscence 0–15.8%, hematoma 1–16%, seroma 1.9–18%) [27]. The most frequent complications of abdominoplasty are wound dehiscence, seroma, and cellulitis, which, although they prolong postoperative wound healing, generally have a low impact on surgical outcomes. Major complications are rare [27].​​ In our previous study, the complication rate after HELP abdominoplasty was 9.1%, while in the present study, it was 11.8%. Thorough evaluation of the abdominal wall before herniorrhaphy is essential, particularly to recognize slim patients with symptomatic RD. The aesthetic outcome should also be considered in the comprehensive management of these patients.

The limitations of this study include the small sample size and the fact that it reflects the experience of a single surgeon. No imaging was done for postoperative evaluation, as it was not clinically needed. All patients have had the possibility of contacting the primary surgeon in case of any suspected recurrence.

## Conclusions

The patient group with primary midline hernias is not homogeneous. This study is not applicable to patients with massive weight loss or obesity; however, it highlights the distinct mechanical issues observed in rectus diastasis in normal-weight patients. These normal-weight women who develop a midline hernia after pregnancy, a thorough evaluation of the entire anterior abdominal wall is essential. When concomitant RD is present, the primary surgical target should be the loose, excess, low-quality linea alba and fascia, with the hernia being a secondary concern. In this subgroup, mesh-augmented herniorrhaphy may not be necessary, if the underlying physiological and biomechanical issues causing core instability, back pain, and hernias, are effectively addressed. Mesh-augmented hernia repair for small hernias may result in suboptimal aesthetic and functional outcomes if the unaddressed rectus diastasis continues to allow abdominal protrusion. Subsequent RD repair after mesh placement can pose significant technical challenges.

Our small series suggests that multi-layer plication and preservation of the midline, along with herniorrhaphy without mesh, result in good long-term outcomes without recurrence of RD or hernia.

## Data Availability

De-identified datasets may be shared upon reasonable request from the corresponding author.
